# High-intensity corneal collagen crosslinking with riboflavin and UVA in rat cornea

**DOI:** 10.1371/journal.pone.0179580

**Published:** 2017-06-23

**Authors:** Yirui Zhu, Peter S. Reinach, Hanlei Zhu, Qiufan Tan, Qinxiang Zheng, Jia Qu, Wei Chen

**Affiliations:** School of Ophthalmology and Optometry and Eye Hospital, Wenzhou Medical University, Zhejiang, China; Oklahoma State University Center for Health Sciences, UNITED STATES

## Abstract

Corneal collagen cross-linking (CXL) halts human corneal ectasias progression by increasing stromal mechanical stiffness. Although some reports describe that this procedure is effective in dealing with some infectious and immunologic corneal thinning diseases, there is a need for more animal models whose corneal thickness more closely resemble those occurring in these patients. To meet this need, we describe here high-intensity protocols that are safe and effective for obtaining CXL in rat corneas. Initially, a range of potentially effective UVA doses were evaluated based on their effectiveness in increasing tissue enzymatic resistance to dissolution. At UVA doses higher than a threshold level of 0.54 J/cm^2^, resistance to enzymatic digestion increased relative to that in non-irradiated corneas. Based on the theoretical threshold CXL dose, a CXL regimen was established in which the UVA tissue irradiance was 9 mW/cm^2^, which was delivered at doses of either 2.16, 2.7 or 3.24 J/cm^2^. Their dose dependent effects were evaluated on ocular surface morphological integrity, keratocyte apoptotic frequency, tissue thickness and endothelial cell layer density. Doses of 2.16 and 2.7 J/cm^2^ transiently decreased normal corneal transparency and increased thickness. These effects were fully reversed after 14 days. In contrast, 3.24 J/cm^2^ had more irreversible side effects. Three days after treatment, apoptotic frequency in the CXL-2.16 group was lower than that at higher doses. Endothelial cell losses remained evident only in the CXL-3.24 group at 42 days posttreatment. Stromal fiber thickening was evident in all the CXL-treated groups. We determined both the threshold UVA dose using the high-intensity CXL procedure and identified an effective dose range that provides optimal CXL with minimal transient side effects in the rat cornea. These results may help to provide insight into how to improve the CXL outcome in patients afflicted with a severe corneal thinning disease.

## Introduction

Corneal collagen cross-linking with riboflavin and UVA is the preferred treatment modality to strengthen biomechanical corneal properties and halt keratoconus progression as well as stabilize ectasia [1–3]. The standard CXL procedure firstly introduced by Wollensak et al [1] for patient use includes delivering UVA irradiation of 3 mW/cm^2^ for 30 min (i.e. 5.4 J/cm^2^) in combination with applying a 0.1% riboflavin solution as long as the corneal thickness is at least 400 μm [4]. On the other hand, the high-intensity (accelerated) CXL procedure applies the same dose as that used in the standard procedure by increasing the intensity even though UVA irradiance time is shortened. This modification increases patient comfort.

Although CXL is still a relatively new procedure extensively used in clinical practice, it is still undergoing further development for improving its outcome with minimal side effects. Such a need is apparent because in some clinical cases, involving infectious and non-infectious stromal melting, corneal perforation and sterile infiltrates appear after CXL [5–8]. Accordingly, improvement of the CXL procedure is supported by determining the effects that it has on ocular surface cellular and molecular properties as well as immune privilege.

The safety and potential phototoxic effects of different UVA doses in the CXL procedure on the endothelial layer have been described in ex vivo porcine corneas [3] and in vivo rabbits[9, 10]. F Wang [11] firstly used a mouse model in which UVA irradiation combined with riboflavin induced stromal apoptosis in vitro. In this study, the UVA intensity was reduced below 1.2 mW/cm^2^ whereas a higher riboflavin concentration was applied because their deepithelialized corneal thickness is only 75μm [12]. These changes were effective in preventing loss of the corneal endothelial layer. The Hafezi [13, 14] group analyzed the effects of a fluence range on the biomechanical and morphological characteristics of murine corneas in order to identify UVA intensities that optimize increases in biomechanical strength without exceeding the threshold intensity for inducing endothelial cell layer damage. Even though the results obtained with the in vivo mouse model are informative in defining underlying cellular and molecular pathways activated by CXL treatment, they may differ from those in humans whose corneal thickness is about five-fold greater than in mice.

In some clinical studies, CXL treatment was effective in managing pellucid marginal degeneration [15], infectious keratitis [16] and had the potential to heal corneal ulcers [17, 18]. However, the underlying mechanisms are poorly understood that alleviate the symptoms of infectious and immunologic corneal thinning diseases. The rat is a viable model to deal with these issues since its corneal full thickness is approximately 160 μm which can approach some of the thicknesses reported in human corneal thinning diseases [19]. Another reason for using the rat model is that Bowman’s membrane is absent which mimics what occurs in some human pathological corneal conditions such as infectious ulcers, stromal melting and pellucid marginal degeneration [20]. Furthermore, the rat corneal MHC antigen expression pattern is controllable and similar to that in humans [21]. Accordingly, identifying the appropriate experimental conditions for obtaining a desirable stromal CXL outcome is relevant for performing the high-intensity CXL protocol in patients whose corneas have become infected or shrank due to activation of an immunological response.

We define here the threshold UVA dosages for obtaining an effective and safe corneal CXL outcome with the high intensity UVA protocol in rats. Efficacy of CXL was evaluated based on increases in the resistance to enzymatic digestion and extent of stromal apoptosis. Safety was evaluated based on extent of recovery of endothelial cell layer and ocular surface integrity and intactness.

## Materials and methods

### Theoretical CXL parameters

In order to predict a safe and effective high-intensity CXL protocol for the rat model, the standard CXL protocol UVA dose and duration were reduced since the rat thickness is much less than that in the human. Two equations derived from Lambert-Beer law were used for this purpose [14]. The human corneal endothelial cell layer UVA damage threshold was set at 0.18 mW/cm^2^ [22]. Based on this analytical approach, a 0.22% riboflavin solution in conjunction with a UVA dose of 2.2 J/cm^2^ were predicted as being safe and effective.

### Study design

All procedures were approved by the Animal Care and Ethics Committee of Wenzhou Medical University, Zhejiang, China, and adhered to the (ARVO) Statement for the Use of Animals in Ophthalmic and Vision Research (S2 File). Eight-to-10-week-old male Sprague-Dawley rats (n = 108) were randomly separated into two sets and exposed to UVA doses according to the Bunsen-Roscoe law of reciprocity between irradiation intensity and duration shown in Tables 1 and 2.

**Table 1:** Identification of a threshold UVA dose mediating CXL.

Group	Intensity, mW/cm2	Irradiation Time, sec	Irradiation Dose, J/cm2
CXL-3.24	9	360	3.24
CXL-2.7	9	300	2.7
CXL-2.16	9	240	2.16
CXL-1.62	9	180	1.62
CXL-1.08	9	120	1.08
CXL-0.54	9	60	0.54
CXL-0.27	9	30	0.27
CXL-0.18	9	20	0.18
CXL-0.09	9	10	0.09
Control	-	-	-

**Table 2:** UVA doses evaluated for efficacy and safety.

Group	Intensity, mW/cm2	Irradiation Time,sec	Irradiation Dose, J/cm2
Control	-	-	-
CXL-2.16	9	240	2.16
CXL-2.7	9	300	2.7
CXL-3.24	9	360	3.24

Both sets composed of 108 rats were exposed to 9 mW/cm^2^ and 0.22% riboflavin. For the first part of the study, 60 rats were equally separated into 10 groups. The UVA irradiation dose was consecutively reduced through steps indicated in Table 1 to determine the CXL threshold level. Resistance to enzymatic digestion of the rat cornea was used as an index of CXL efficacy. This assessment is based on the time required to completely dissolve the tissue. Increases in efficacy are associated with prolongation of time required for total tissue dissolution due to its compaction. This occurs because compaction masks proteolytic sites from enzymatic access [23, 24]. In another assessment using 12 rats from each group, UVA doses were increased in steps above a theoretical maximum UVA threshold to determine their individual effects on the ocular surface, endothelial layer integrity and intactness, as well as keratocyte apoptosis.

### Corneal cross-linking procedure

**Fig 1:**
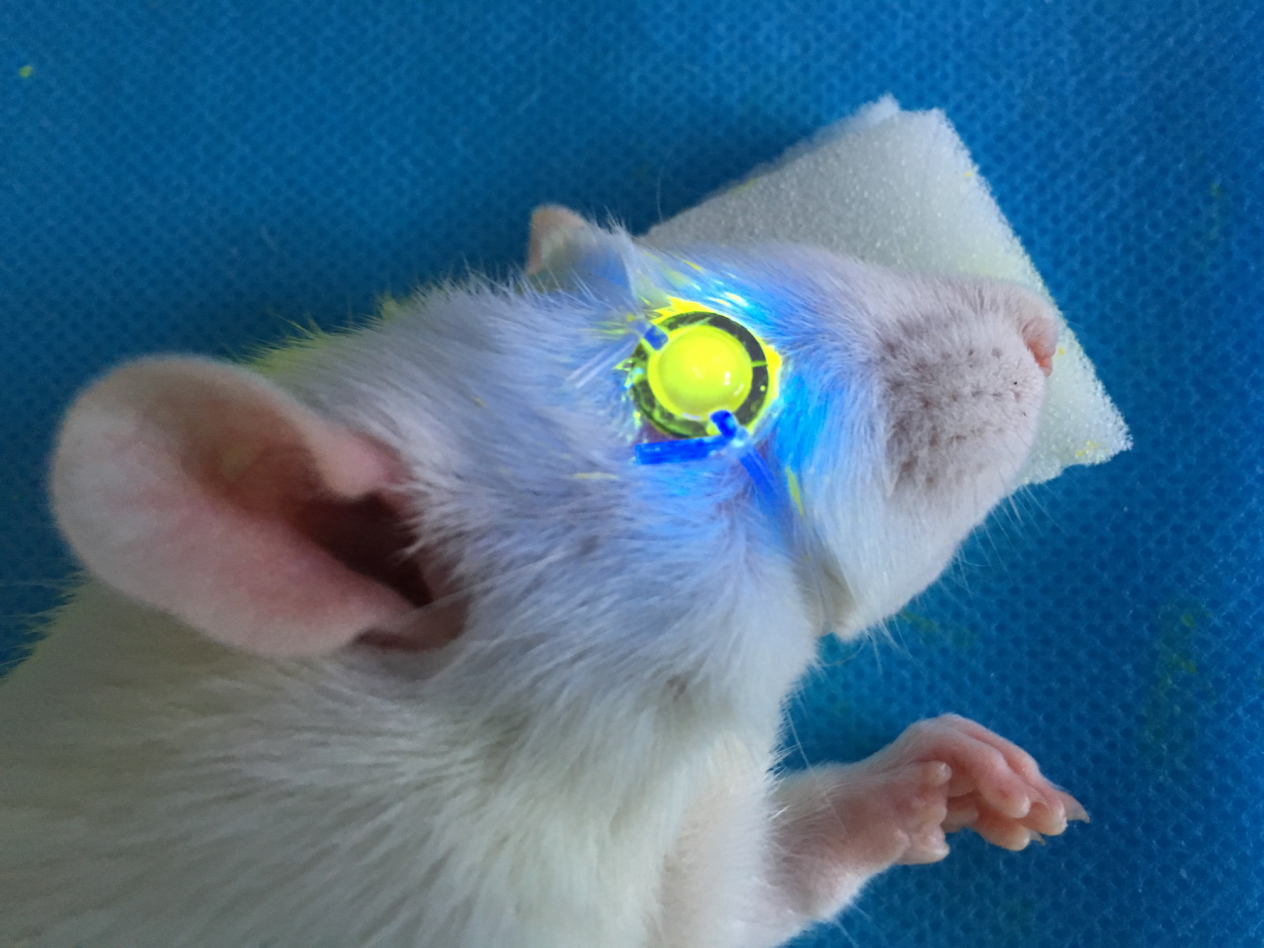
Delimiting UVA/riboflavin rat eye exposure. UVA irradiation was restricted to the central cornea by placing atop the ocular surface a metal ring having an 8 mm diameter opening. This precaution protects the limbal area from being exposed to UVA irradiation.

Anesthesia was administered by intraperitoneal injection of ketamine hydrochloride (50–75 mg/kg), xylazine hydrochloride (5 mg/kg), and proparacaine eye drops were topically applied. Corneas were mechanically de-epithelialized spanning a 5 mm diameter. Isotonic 0.22% riboflavin solution eyedrops [10 mg riboflavin in 4.5 ml of 20% dextran T-500 solution (Peschke GmbH, Nürnberg, Germany)] were instilled every 3 min for 30 min. Riboflavin penetration into the anterior chamber was evaluated with slit lamp biomicroscopy using blue light. The limbus and conjunctiva were protected by covering this region with a metal ring affixed to the rat head, designed and produced by the authors, which left only the de-epithelialized cornea exposed to UVA irradiation. The right eye was irradiated at a surface intensity of 9 mW/cm^2^ for varying times listed in Table 1 (UV-360, Medical Photon Corp, Xiamen, China; Fig 1). The indicated riboflavin solution was reapplied topically to the corneal surface every 5 min. Following CXL, Tobramycin eye drops (Tobradex; Alcon Laboratories, Inc) were administered four times daily until the corneal epithelial wound healed.

### Enzymatic digestion

Following CXL treatment, a 6 mm full-thickness tissue biopsy was trephined from the center of each cornea. The corneal disks (6 from each group) were placed in individual tubes containing 0.5 mL 0.3% collagenase solution (active agent: Clostridium histolyticum) (Sigma-Aldrich, Shanghai, China) and diluted in Dulbecco’s Phosphate Buffered Saline (DPBS, Sigma-Aldrich) and incubated at 37°C in a bath placed on a platform rotating 175 times per min [24]. The extent of tissue digestion was monitored every 30 min for the first 2 h and after that every 15 min until the tissue had been solubilized.

### Ocular surface evaluation

After CXL, corneas were examined every day with a slit lamp. Inflammatory index values were assigned based on summing the scores of the different described parameters used for this purpose on postoperative days 3, 7, 14, 21, 28 and 42 [25]. Inflammatory index values were obtained based on the following scale: Ciliary hyperemia (absent, 0; present but extending less than 1mm, 1; hyperemia extending between 1 and 2 mm, 2; present and extending more than 2 mm, 3); central corneal edema (absent, 0; present with visible iris details, 1; present without visible iris details, 2; present without visible pupil, 3); peripheral corneal edema (absent, 0; present with visible iris details 1; present without visible iris details, 2; present without visible iris, 3).

### Anterior segment optical coherence tomography

A described custom built, high speed, ultra-high resolution spectral domain optical coherence tomograph (UHR-OCT) postoperatively assessed corneal CXL outcome on day 3, 7, 14, 28 and 42 [26, 27].

### In vivo confocal microscopy

The anesthetized rats (6 rats in each group) were examined pretreatment and post-CXL from day 1 to 42 and compared to control eyes with both a scanning laser IVCM and the Heidelberg Retina Tomograph Ⅲ (HRT Ⅲ), Rostock Cornea Module (RCM; Heidelberg Engineering, GmBH, Germany). The mean corneal thickness was calculated by URH-OCT and in vivo confocal microscopy based on the depth difference between the most superficial and the deepest corneal structures. Endothelial cell densities were evaluated using a built-in software program that expressed the number of marks in terms of cells per square millimeter.

### Immunofluorescent staining

Keratocyte damage was evaluated with a fluorescence-based TUNEL assay (In Site Cell Death Detection Kit; Roche Applied Science, Indianapolis, IN) according to the manufacturer’s instructions. The images were photographed with a laser scanning confocal microscope (LSM710; Zeiss coupled to a krypton-argon and He-Ne laser; Carl Zeiss Meditec, Sartrouville, Germany) at 20 × magnification. The mean number of keratocytes was determined by counting them in five non-overlapping areas (0.1 mm × 0.1 mm) of three separate corneal sections.

### Transmission electron microscopy

Three rats from each group were sacrificed on day 7, The enucleated bisected corneal sections were fixed in 2.5% glutaraldehyde in 0.1 M phosphate buffer (pH 7.4) at 4°C. Samples were sequentially processed in 1% osmium tetroxide for 1 h. The tissues were dehydrated in a graded ethyl alcohol series and embedded in Epoc 812. The ultrathin sections were cut using a RT-7000 (RMC, USA), stained with uranyl acetate and lead citrate, and then examined with a transmission electron microscope (H-7650; Hitachi, Tokyo, Japan) at 80KV voltage.

### Statistical analysis

Data are expressed as mean ± standard error of the mean (SEM) where appropriate. Analyses were performed using SPSS 19.0 software (IBM SPSS Statistics, IBM Corporation, Chicago, IL). The p value was determined using the Mann-Whitney U test and the Kruskal-Wallis test. p < 0.05 was considered statistically significant.

## Results

### UVA dose dependent effects on resistance to enzymatic tissue dissolution

At each UVA dose above a threshold of 0.54 J/cm^2^ provided by exposing corneas to 9 mW/cm^2^ for 1 min, enzymatic resistance to tissue dissolution increased based on prolongation of the time required for this process to go to completion (Table 3, p < 0.01). On the other hand, exposure to this UVA intensity for only either 10, 20 or 30 sec did not extend the time required for enzymatic digestion to times greater than those needed to dissolve control corneas.

**Table 3:** Dependence of enzymatic dissolution time on UVA dose.

Group	Average time (min)	Standard deviation	p (Group to Control)
CXL-3.24	346	9	0.004
CXL-2.7	328	7	0.004
CXL-2.16	312	5	0.004
CXL-1.62	307	5	0.004
CXL-1.08	282	5	0.004
CXL-0.54	246	3	0.004
CXL-0.27	239	3	0.063
CXL-0.18	237	4	0.259
CXL-0.09	235	4	0.684
Control	235	3	-

Six corneal samples per group. p = significance level between group and control.

### Clinical evaluation

**Fig 2:**
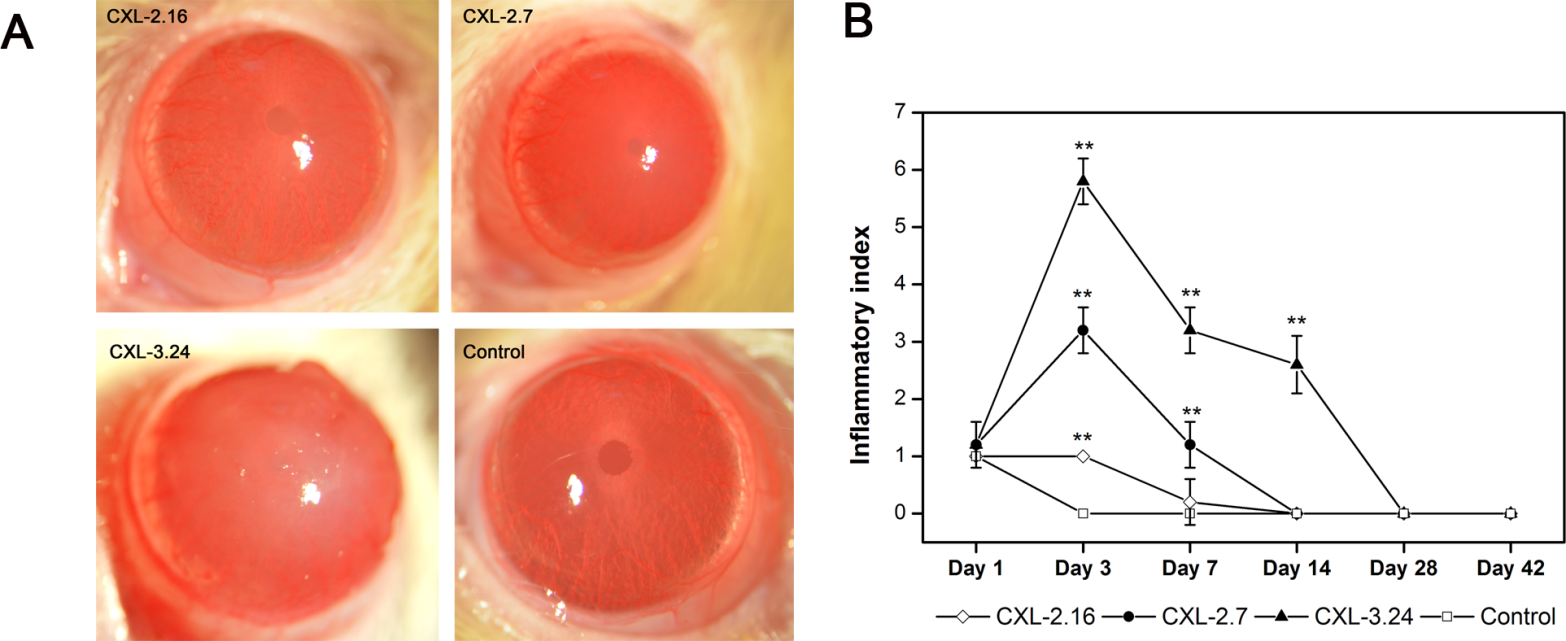
Differential effects of CXL treatment on clinical signs. (A) Representative slit lamp images showing differences between the 4 different groups on day 7 after CXL-treatment. (B) Time dependent changes in inflammatory index among the 4 groups from day 1 to 42 (Data are presented as Mean ±(SEM, CXL groups versus control group; n = 6, **p < 0.01).

**Fig 3:**
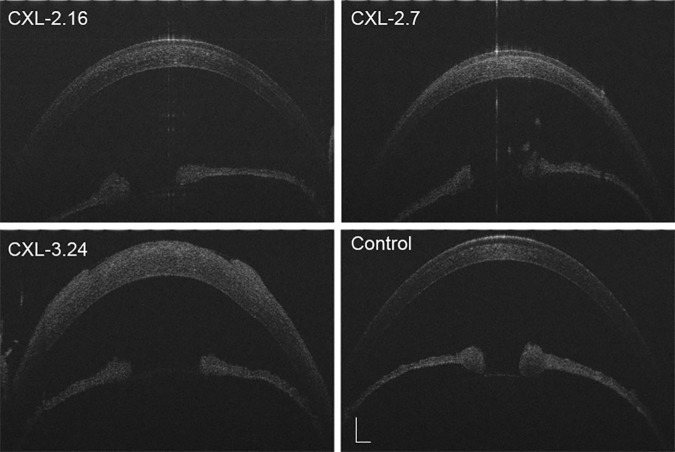
Anterior segment optical coherence tomography. UHR-OCT scan visualized the anterior segment integrity at 7 days posttreatment. It appeared normal except for mild corneal edema after receiving doses of either 2.16 or 2.7 J/cm^2^. Significant corneal edema and disrupted epithelial intactness and discontinuity in the central area developed in the CXL-3.24 group (n = 6, Scale bar = 500 μm).

**Fig 4:**
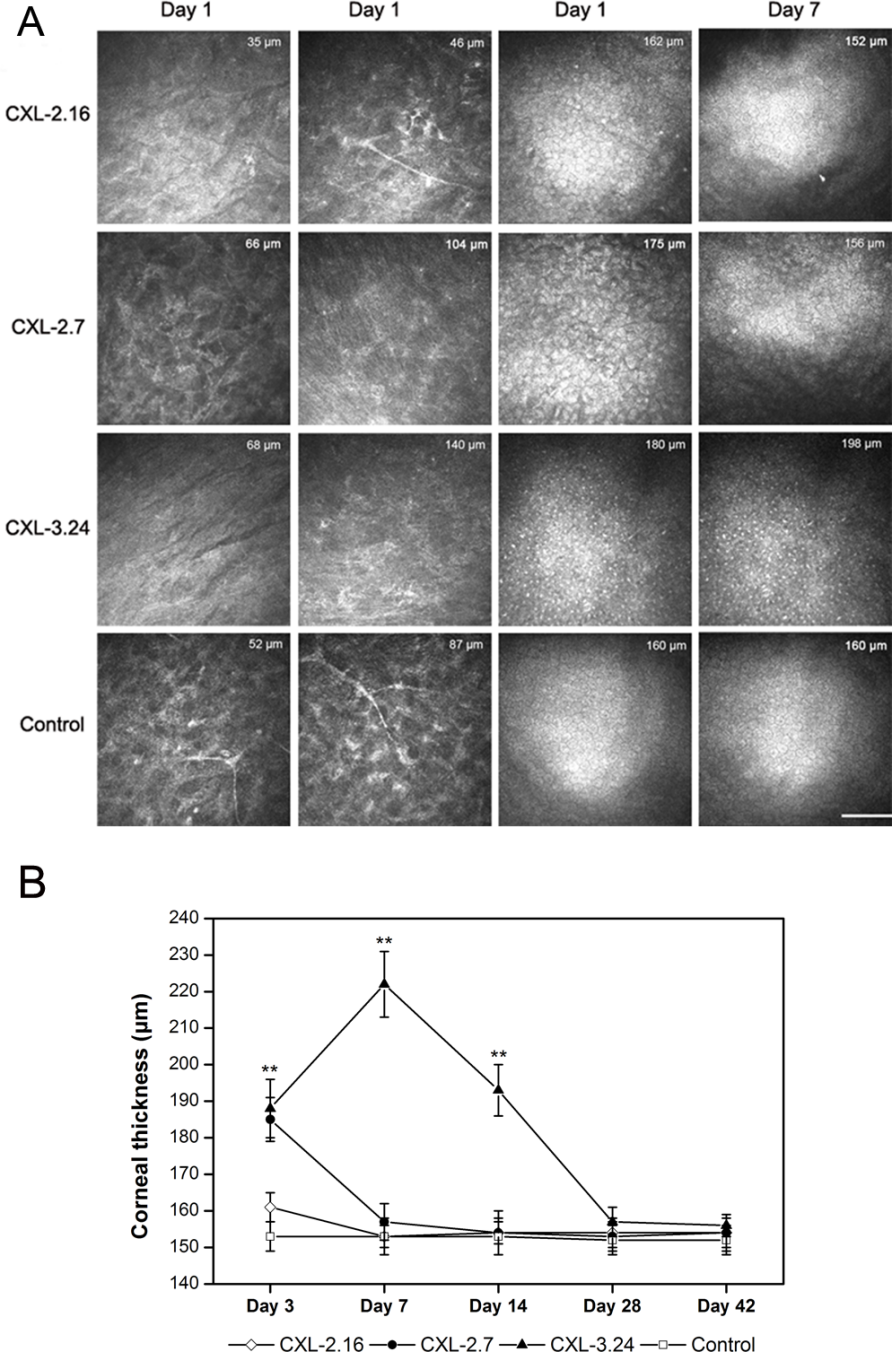
In vivo confocal microscopy scan of CXL rat corneas. (A) Representative confocal microscopy images showing differences between the 4 different groups in anterior and posterior stroma and endothelial cell layer on day 1 and 7 after CXL-treatment. The thickness measured between the uppermost corneal surface and the endothelial layer underside is indicated on each image (n = 6. scale bar = 100 μm). (B) Mean corneal thickness calculated using in vivo confocal microscopy and UHR-OCT after CXL treatment (n = 6).

Slit lamp examination revealed mild corneal edema and loss of epithelial continuity and breaching of the barrier function in the central area of each CXL-treated group on posttreatment day 1. In the CXL-2.16 and 2.7 groups, corneal transparency was restored in all cases after 7 days whereas they remained edematous and opalescent in the CXL-3.24 group, which is consistent with disappearance of the endothelial cell layer (Fig 2A). At 42 days post CXL, only those corneas in the CXL-2.16 and 2.7 groups appeared transparent. Compared to the control group, at day 3 after CXL the inflammatory index increased in all the CXL-treated groups. In the CXL-2.16 and CXL-2.7 groups, these effects reversed back to their baseline values at day 7 and day 14 respectively, whereas in the CXL-3.24 group its level remained high until day 28 (p < 0.01, Fig 2B). Seven days after CXL, UHR-OCT showed that in the CXL-2.16 and 2.7 groups the upper corneal epithelial layers regained cell to cell contact. However, the cells appeared edematous which disrupted their margin integrity in the CXL-3.24 group without undergoing recovery (Fig 3). At day 28, the CXL-3.24 stroma was compacted along with scar formation in the central region. In this group, on day 1 after performing the CXL procedure, confocal microscopy examination revealed stromal lacunar edematous regions associated with keratocyte losses in all the CXL-treated groups (Fig 4A). On day 14, stromal keratocyte proliferation was observed in all three groups.

### Corneal thickness

The mean corneal thickness was 153±4 μm in the control and treated groups prior to treatment. In the CXL 2.7 and 3.24 J/cm^2^ treated groups, their thicknesses increased significantly to reach 180 and 200 μm, respectively, on day 3 (p < 0.01). From day 7 onwards, neither the CXL 2.7 J/cm^2^ nor 2.16 J/cm^2^ groups underwent any more significant thickness changes and had reversed to values similar to those in untreated corneas. In the CXL-3.24 group, its thickness remained significantly greater than the control until day 14 (p < 0.01). By day 28 posttreatment, it decreased to baseline value. In the 2.16 J/cm^2^ group, the thickness tended to increase by day 3 without reaching significance and it returned to normal by day 7 (Fig 4B).

### Keratocyte density losses

**Fig 5:**
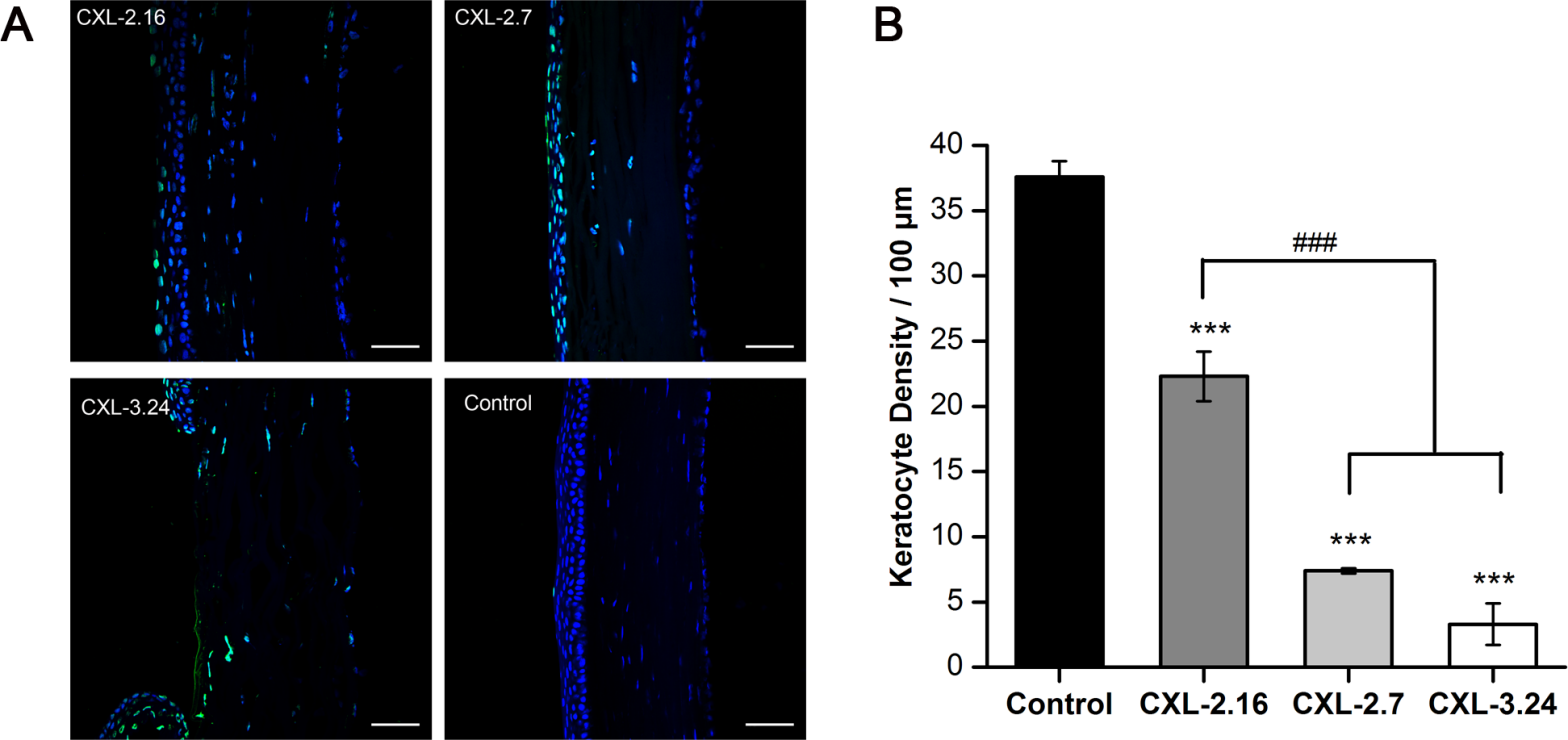
CXL-induced apoptosis and keratocyte density. (A) Apoptosis of the rat keratocytes (green, positive TUNEL staining) are present in the CXL-treated areas and control cornea on day 3. The control cornea had a normal DAPI staining pattern. In the cross-linked corneas, some keratocyte were apoptotic. Apparent endothelial layer damage was detected in the CXL-3.24 group. (B) Significant difference was evident between keratocyte density in CXL-treated and control corneas on day 3. The keratocyte counts significantly decreased in the CXL-2.7 and 3.24 groups compared with the CXL-2.16 group (### p < 0.01 versus CXL-treated groups, *** p < 0.001 versus control group, respectively; Data are presented as Mean ± SEM, n = 3. scale bars = 50 μm).

In the CXL-2.16 group, the integrity of the mid-posterior stromal region remained essentially unchanged compared to that in the control group. In contrast, with the CXL 2.7 group, most of the keratocytes were apoptotic at all depths in the stroma on day 3 after CXL. This trend became more apparent in the CXL-3.24 group (Fig 4). In the CXL-2.16 group, TUNEL-positive cells were localized to the superficial anterior stroma on posttreatment day 3. Apoptotic frequency throughout the stroma became progressively more evident in the CXL-2.7 and CXL-3.24 groups. In the latter group, the few remaining endothelial cells were apoptotic (Fig 5A). On the other hand by day 3 after CXL treatment, keratocyte frequencies in the three CXL treated groups were significantly lower than those in the untreated cornea (p < 0.001). The keratocyte counts significantly decreased in the CXL-2.7 and 3.24 groups compared with the CXL-2.16 group (p < 0.001, Fig 5B).

### Endothelial cell density recovery

Table 4 shows the time dependent recovery of endothelial cell density as a function of CXL dose. In the CXL 3.24 group, the endothelial cells became edematous from days 1 to 7 followed by complete endothelial cell disappearance. Endothelial damage from day 14 to 42 persisted and endothelial cell densities were still significantly less than in the control group. The CXL-2.7-induced decline in endothelial cell density fully reversed by day 28. Irradiation of 2.16 J/cm^2^ seemed not to inflict significant endothelial cell damage (Fig 4A, Table 4).

**Table 4:** Time dependence of central endothelial cell density recovery after different CXL treatments.

Days	Control cells/mm^2^	CXL-2.16 cells/mm^2^	CXL-2.7 cells/mm^2^	CXL-3.24 cells/mm^2^
1	2706 **±** 44	2679 **±** 51	2220 **±** 83**	0***
7	2718 **±** 36	2703 **±** 44	2282 **±** 47**	0***
14	2740 **±** 57	2734 **±** 32	2378 **±** 31**	327 **±** 43***
28	2719 **±** 41	2732 **±** 45	2656 **±** 49	1217 **±** 33**
42	2735 **±** 37	2746 **±** 38	2723 **±** 36	1753 **±** 57**

SEM, standard error of the mean.

**p < 0.01

***p < 0.001 compare to control group.

### Ultrastructural changes

**Fig 6:**
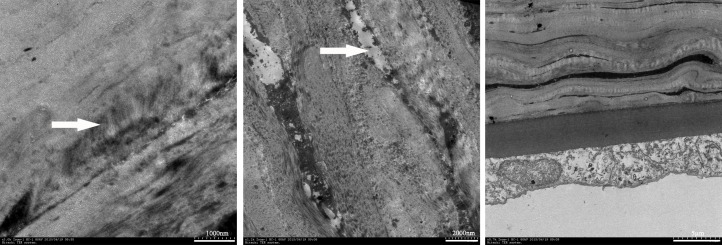
CXL-induced changes in stromal and endothelium ultrastructure on day 7. The stroma had a compacted collagenous matrix (Left arrow) containing degenerative changes in keratocytes across the stroma in all CXL-treated groups (Middle arrow). No such defects were detected in the posterior stroma and endothelium of the CXL-2.16 group (n = 3).

The micrographs shown in Fig 6 indicate that the anterior stromal collagenous architecture was a more compacted matrix than that in the untreated corneas with some apoptotic cell figures in all CXL-treated corneas. The endothelial layer was relatively free of them in the CXL-2.16 group.

## Discussion

We determined the threshold UVA dose needed to induce collagen CXL as well as the optimal UVA dose range whose side effects are minimal and reversible in rat corneas. Such a characterization in this complementary novel model may provide insight into how to apply the high intensity CXL protocol to treat pathological conditions causing thinning of this tissue to even less than 200 μm in a clinical setting. We identified a threshold UVA dose of 0.54 J/cm^2^ because which represents the lowest dose for increasing collagen CXL. In associating the increases in enzymatic resistance with reversibility of damaging side effects on corneal integrity, we found that CXL using 9 mW/cm^2^ for 4 min (2.16 J/cm^2^) with 0.22% riboflavin is appropriate for obtaining a safe and effective CXL outcome. At UVA doses between 2.16 and 2.7 J/cm^2^, corneal transparency was maintained, while 3.24 J/cm^2^ is too high a dose because losses in central endothelial cell density were more extensive and irreversible. Therefore, a UVA dose of 2.7 J/cm^2^ is the maximum dose for inducing CXL without eliciting irreversible losses in endothelial cell density and stromal integrity in the rat.

The association between increases in UVA doses and enzymatic resistance to tissue dissolution was used to evaluate collagen CXL efficiency [23, 28–30]. Taking into account the differences in corneal thickness, the threshold UVA dose of 0.54 J/cm^2^ in the rat is in accord with the reported values in the mouse and human of 0.09 [13] and 5.4 J/cm^2^ [1], respectively whose values reflect differences in corneal thicknesses from 100 to 530 μm, respectively. It has been suggested that greater oxygen availability with thinner corneas helps explain why lower UVA doses are safe and effective in these animal models [13].

Assessment of non-specific stromal effects showed that UVA dose dependent increases occurred in the ocular inflammatory index and stromal apoptotic frequency. UVA irradiation of 2.16 J/cm^2^ seemed not to inflict significant endothelial cell damage whereas the inflammatory index and corneal thickness tended to increase by day 3. Corneal stroma edema and hyperemia may explained by cornea immune responses induced after CXL treatment [31]. Even though in all cases the increases in the inflammatory index were fully reversible 28 days after treatment, the corneas treated with 3.24 J/cm^2^ failed to become transparent. In vivo confocal microscopy showed that with a UVA dose of 2.16 J/cm^2^ keratocyte damage was limited to the superficial stroma. In contrast, with 2.7 J/cm^2^ this damage was more evident and more extensive in the stroma. At the highest dose of 3.24 J/cm^2^, epithelial denudation was irreversible. These alterations are consistent with those seen in electron micrographs and TUNEL assays. Furthermore these effects are similar to the described structural changes and keratocyte losses with the standard CXL procedure in humans [32] and rabbits [33]. In transmission electron micrographs of CXL treated rabbit and human corneas, keratocyte losses due to apoptosis were accompanied by anterior stromal collagen fiber compaction [34–36]. We also found similar collagen structural reorganization in the CXL-treated anterior stroma. Taken together, a UVA dose no larger than 2.7 J/cm^2^ is relatively safe since all of its non-selective effects were reversible after no more than 14 days.

The standard CXL protocol is contraindicated in patients whose corneas are thinner than 400 μm because of endothelial cytotoxic effects. The results obtained with the CXL-2.16 group in this study may have relevance for applying CXL in diseased corneas undergoing thinning since endothelial cell density did not decrease. A slight decrease in endothelial density accompanied by some edema occurred in the CXL-2.7 group. Density initially fell to 2220**±**83 cells/mm^2^ on posttreatment day 1, but this decline reversed since in vivo confocal microscopy clearly showed that endothelial cell repopulation occurred which was accompanied by restoration of control endothelial cell layer density and a normal hexagonal cobblestone morphological arrangement 28 days after CXL treatment. This reversal agrees with reports describing that the rat endothelium undergoes time dependent functional recovery based on eventual restoration of corneal transparency accompanied by increases in proliferation and migration after surgical damage [37]. In contrast, no endothelial cells were detected 7 days after CXL-3.24 CXL treatment. Even after 42 days recovery was incomplete indicating that this UVA dose is too injurious and ill advised. These results suggest that the maximal UVA dose for high-intensity CXL should not exceed 2.7 J/cm^2^.

With our CXL protocol, irradiation was performed in a centered fashion over an 8 mm diameter area and a metal ring was placed over the limbus to shield it from UVA exposure. This was done to prevent partial irradiation of the limbus and conjunctiva. Such precaution seemed to be effective because epithelial regeneration occurred normally in the absence of any signs of limbal stem cell deficiency.

In conclusion, the high-intensity CXL protocol has a CXL threshold of approximately 0.54 J/cm^2^ in the in vivo rat model. At UVA doses up to approximately 2.7 J/cm^2^ stromal crosslinking occurs with reversible endothelial cell damage and transient declines in keratocyte viability. These results will help to identify other possible risks and ocular immune responses that could compromise the outcome of this procedure if used on patients afflicted with a corneal thinning disease. They may also be helpful in developing new and more efficient CXL protocols to treat human corneal disorders such as infectious corneal ulcers and immunologic corneal thinning diseases.

## Supporting information

S1 File ARRIVE guidelines checklist. (PDF)

S2 File Animal experimental ethical inspection. (PDF)

## References

[1] Wollensak G, Spoerl E, Seiler T. Riboflavin/ultraviolet-a-induced collagen crosslinking for the treatment of keratoconus. Am J Ophthalmol. 2003;135(5):620–7. . 12719068 10.1016/s0002-9394(02)02220-1

[2] Spoerl E, Huhle M, Seiler T. Induction of cross-links in corneal tissue. Exp Eye Res. 1998;66(1):97–103. doi: 10.1006/exer.1997.0410 . 9533835 10.1006/exer.1997.0410

[3] Wollensak G, Spoerl E, Seiler T. Stress-strain measurements of human and porcine corneas after riboflavin-ultraviolet-A-induced cross-linking. J Cataract Refract Surg. 2003;29(9):1780–5. . 14522301 10.1016/s0886-3350(03)00407-3

[4] Spoerl E, Mrochen M, Sliney D, Trokel S, Seiler T. Safety of UVA-riboflavin cross-linking of the cornea. Cornea. 2007;26(4):385–9. doi: 10.1097/ICO.0b013e3180334f78 . 17457183 10.1097/ICO.0b013e3180334f78

[5] Rama P, Di Matteo F, Matuska S, Paganoni G, Spinelli A. Acanthamoeba keratitis with perforation after corneal crosslinking and bandage contact lens use. J Cataract Refract Surg. 2009;35(4):788–91. doi: 10.1016/j.jcrs.2008.09.035 . 19304108 10.1016/j.jcrs.2008.09.035

[6] Angunawela RI, Arnalich-Montiel F, Allan BD. Peripheral sterile corneal infiltrates and melting after collagen crosslinking for keratoconus. J Cataract Refract Surg. 2009;35(3):606–7. doi: 10.1016/j.jcrs.2008.11.050 . 19251159 10.1016/j.jcrs.2008.11.050

[7] Gokhale NS, Vemuganti GK. Diclofenac-induced acute corneal melt after collagen crosslinking for keratoconus. Cornea. 2010;29(1):117–9. doi: 10.1097/ICO.0b013e3181a06c31 . 19907307 10.1097/ICO.0b013e3181a06c31

[8] Koller T, Mrochen M, Seiler T. Complication and failure rates after corneal crosslinking. J Cataract Refract Surg. 2009;35(8):1358–62. doi: 10.1016/j.jcrs.2009.03.035 . 19631120 10.1016/j.jcrs.2009.03.035

[9] Wollensak G, Spoerl E, Wilsch M, Seiler T. Endothelial cell damage after riboflavin-ultraviolet-A treatment in the rabbit. J Cataract Refract Surg. 2003;29(9):1786–90. . 14522302 10.1016/s0886-3350(03)00343-2

[10] Schumacher S, Oeftiger L, Mrochen M. Equivalence of biomechanical changes induced by rapid and standard corneal cross-linking, using riboflavin and ultraviolet radiation. Invest Ophthalmol Vis Sci. 2011;52(12):9048–52. doi: 10.1167/iovs.11-7818 . 22025568 10.1167/iovs.11-7818

[11] Wang F. UVA/riboflavin-induced apoptosis in mouse cornea. Ophthalmologica. 2008;222(6):369–72. doi: 10.1159/000151247 . 18698146 10.1159/000151247

[12] Zhang H, Wang L, Xie Y, Liu S, Deng X, He S, et al The measurement of corneal thickness from center to limbus in vivo in C57BL/6 and BALB/c mice using two-photon imaging. Exp Eye Res. 2013;115:255–62. doi: 10.1016/j.exer.2013.07.025 . 23920154 10.1016/j.exer.2013.07.025

[13] Hammer A, Kling S, Boldi MO, Richoz O, Tabibian D, Randleman JB, et al Establishing Corneal Cross-Linking With Riboflavin and UV-A in the Mouse Cornea In Vivo: Biomechanical Analysis. Invest Ophthalmol Vis Sci. 2015;56(11):6581–90. doi: 10.1167/iovs.15-17426 . 26465887 10.1167/iovs.15-17426

[14] Kling S, Hammer A, Conti A, Hafezi F. Corneal Cross-Linking with Riboflavin and UV-A in the Mouse Cornea in Vivo: Morphological, Biochemical, and Physiological Analysis. Transl Vis Sci Technol. 2017;6(1):7 doi: 10.1167/tvst.6.1.7 ; PubMed Central PMCID: PMCPMC5283086. 28149672 10.1167/tvst.6.1.7 PMC5283086

[15] Spadea L. Corneal collagen cross-linking with riboflavin and UVA irradiation in pellucid marginal degeneration. J Refract Surg. 2010;26(5):375–7. doi: 10.3928/1081597X-20100114-03 . 20128533 10.3928/1081597X-20100114-03

[16] Alio JL, Abbouda A, Valle DD, Del Castillo JM, Fernandez JA. Corneal cross linking and infectious keratitis: a systematic review with a meta-analysis of reported cases. J Ophthalmic Inflamm Infect. 2013;3(1):47 doi: 10.1186/1869-5760-3-47 ; PubMed Central PMCID: PMCPMC3671959. 23718849 10.1186/1869-5760-3-47 PMC3671959

[17] Ehlers N, Hjortdal J, Nielsen K, Sondergaard A. Riboflavin-UVA treatment in the management of edema and nonhealing ulcers of the cornea. J Refract Surg. 2009;25(9):S803–6. doi: 10.3928/1081597X-20090813-08 . 19772255 10.3928/1081597X-20090813-08

[18] Iseli HP, Thiel MA, Hafezi F, Kampmeier J, Seiler T. Ultraviolet A/riboflavin corneal cross-linking for infectious keratitis associated with corneal melts. Cornea. 2008;27(5):590–4. doi: 10.1097/ICO.0b013e318169d698 . 18520510 10.1097/ICO.0b013e318169d698

[19] Bawa G, Tkatchenko TV, Avrutsky I, Tkatchenko AV. Variational analysis of the mouse and rat eye optical parameters. Biomed Opt Express. 2013;4(11):2585–95. doi: 10.1364/BOE.4.002585 ; PubMed Central PMCID: PMCPMC3829552. 24312744 10.1364/BOE.4.002585 PMC3829552

[20] Jakus MA. Studies on the cornea. I. The fine structure of the rat cornea. Am J Ophthalmol. 1954;38(1:2):40–53. Epub 1954/07/01. . 13180617

[21] Treseler PA, Sanfilippo F. The expression of major histocompatibility complex and leukocyte antigens by cells in the rat cornea. Transplantation. 1986;41(2):248–52. . 3456178 10.1097/00007890-198602000-00022

[22] Spoerl E, Hoyer A, Pillunat LE, Raiskup F. Corneal cross-linking and safety issues. Open Ophthalmol J. 2011;5:14–6. doi: 10.2174/1874364101105010014 ; PubMed Central PMCID: PMCPMC3052642. 21399770 10.2174/1874364101105010014 PMC3052642

[23] Spoerl E, Wollensak G, Seiler T. Increased resistance of crosslinked cornea against enzymatic digestion. Curr Eye Res. 2004;29(1):35–40. doi: 10.1080/02713680490513182 . 15370365 10.1080/02713680490513182

[24] Kanellopoulos AJ, Asimellis G, Salvador-Culla B, Chodosh J, Ciolino JB. High-irradiance CXL combined with myopic LASIK: flap and residual stroma biomechanical properties studied ex-vivo. Br J Ophthalmol. 2015;99(6):870–4. doi: 10.1136/bjophthalmol-2014-306411 . 25795914 10.1136/bjophthalmol-2014-306411

[25] Chen Y, Yang W, Zhang X, Yang S, Peng G, Wu T, et al MK2 inhibitor reduces alkali burn-induced inflammation in rat cornea. Sci Rep. 2016;6:28145 doi: 10.1038/srep28145 ; PubMed Central PMCID: PMCPMC4916419. 27329698 10.1038/srep28145 PMC4916419

[26] Wang JH, Jiao SL, Ruggeri M, Abou Shousha M, Chen Q. In Situ Visualization of Tears on Contact Lens Using Ultra High Resolution Optical Coherence Tomography. Eye & Contact Lens-Science and Clinical Practice. 2009;35(2):44–9. doi: 10.1097/ICL.0b013e31819579f2 19265323 10.1097/ICL.0b013e31819579f2 PMC3397174

[27] Lian Y, Shen M, Jiang J, Mao X, Lu P, Zhu D, et al Vertical and horizontal thickness profiles of the corneal epithelium and Bowman's layer after orthokeratology. Invest Ophthalmol Vis Sci. 2013;54(1):691–6. doi: 10.1167/iovs.12-10263 . 23221070 10.1167/iovs.12-10263

[28] Aldahlawi NH, Hayes S, O'Brart DP, Akhbanbetova A, Littlechild SL, Meek KM. Enzymatic Resistance of Corneas Crosslinked Using Riboflavin in Conjunction With Low Energy, High Energy, and Pulsed UVA Irradiation Modes. Invest Ophthalmol Vis Sci. 2016;57(4):1547–52. doi: 10.1167/iovs.15-18769 . 27046119 10.1167/iovs.15-18769 PMC5321166

[29] Aldahlawi NH, Hayes S, O'Brart DP, Meek KM. Standard versus accelerated riboflavin-ultraviolet corneal collagen crosslinking: Resistance against enzymatic digestion. J Cataract Refract Surg. 2015;41(9):1989–96. doi: 10.1016/j.jcrs.2015.10.004 ; PubMed Central PMCID: PMCPMC4670830. 26603408 10.1016/j.jcrs.2015.10.004 PMC4670830

[30] Kanellopoulos AJ, Loukas YL, Asimellis G. Cross-Linking Biomechanical Effect in Human Corneas by Same Energy, Different UV-A Fluence: An Enzymatic Digestion Comparative Evaluation. Cornea. 2016;35(4):557–61. doi: 10.1097/ICO.0000000000000758 . 26845317 10.1097/ICO.0000000000000758

[31] Kolozsvari BL, Berta A, Petrovski G, Mihaltz K, Gogolak P, Rajnavolgyi E, et al Alterations of tear mediators in patients with keratoconus after corneal crosslinking associate with corneal changes. PLoS One. 2013;8(10):e76333 doi: 10.1371/journal.pone.0076333 ; PubMed Central PMCID: PMCPMC3790710. 24124547 10.1371/journal.pone.0076333 PMC3790710

[32] Mazzotta C, Hafezi F, Kymionis G, Caragiuli S, Jacob S, Traversi C, et al In Vivo Confocal Microscopy after Corneal Collagen Crosslinking. Ocul Surf. 2015;13(4):298–314. doi: 10.1016/j.jtos.2015.04.007 . 26142059 10.1016/j.jtos.2015.04.007

[33] Hovakimyan M, Guthoff R, Knappe S, Zhivov A, Wree A, Kruger A, et al Short-term corneal response to cross-linking in rabbit eyes assessed by in vivo confocal laser scanning microscopy and histology. Cornea. 2011;30(2):196–203. doi: 10.1097/ICO.0b013e3181e16d93 . 20861724 10.1097/ICO.0b013e3181e16d93

[34] Dhaliwal JS, Kaufman SC. Corneal collagen cross-linking: a confocal, electron, and light microscopy study of eye bank corneas. Cornea. 2009;28(1):62–7. doi: 10.1097/ICO.0b013e31818225c3 . 19092408 10.1097/ICO.0b013e31818225c3

[35] Messmer EM, Meyer P, Herwig MC, Loeffler KU, Schirra F, Seitz B, et al Morphological and immunohistochemical changes after corneal cross-linking. Cornea. 2013;32(2):111–7. doi: 10.1097/ICO.0b013e31824d701b . 22580432 10.1097/ICO.0b013e31824d701b

[36] Wollensak G, Wilsch M, Spoerl E, Seiler T. Collagen fiber diameter in the rabbit cornea after collagen crosslinking by riboflavin/UVA. Cornea. 2004;23(5):503–7. . 15220736 10.1097/01.ico.0000105827.85025.7f

[37] Schwartzkopff J, Bredow L, Mahlenbrey S, Boehringer D, Reinhard T. Regeneration of corneal endothelium following complete endothelial cell loss in rat keratoplasty. Mol Vis. 2010;16:2368–75. ; PubMed Central PMCID: PMCPMC2994736. 21139971 PMC2994736

