# Supplementary material for: High-intensity corneal collagen crosslinking with riboflavin and UVA in rat cornea
Source: PLoS One. 2017 Jun 23;12(6):e0179580. doi: 10.1371/journal.pone.0179580 (PMC5482453; doi:10.1371/journal.pone.0179580)
Supplement: S2 File — (PDF) [file pone.0179580.s002.pdf]

温州医科大学实验动物中心动物实验伦理审查表

The Tab of Animal Experimental Ethical Inspection of Laboratory Animal Centre, Wenzhou Medical University

批准编号(ID Number): wydw2015-0192

申请人填写的相关信息 (Related information filled by applicant)

申请单位(Name of organization): 温州医科大学眼视光学院 School of Ophthalmology and Optometry and Eye Hospital, Wenzhou Medical University

申请人学历 (Education of applicant): 硕士 Master	技术职称 (Professional title):

实验名称(Experiment title): 高能量紫外线核黄素交联对大鼠角膜作用的实验研究 A rat model of high-intensity corneal collagen cross-linking with riboflavin and UVA

项目来源(Project sources): 国家自然科学基金

National Natural Science Foundation of China (81170820 and 81470605 to Wei Chen).

实验目的(Aim of experiment): 建立紫外线核黄素高能量角膜交联的大鼠模型

To develop a modified high-intensity corneal collagen cross-linking for use in rats

实验动物使用许可证号(Number of the using of Laboratory Animal):

SYXK（浙）2015-0009

拟进动物情况

动物来源(Source of animal): 温州医科大学实验动物中心 Laboratory Animal Centre, Wenzhou Medical University

品种品系(Species or strain): Sprague-Dawley 等级(Grade): CL 规格(Specifications): 200-220g

数量(Number): 90 只 (♀ 只; ♂ 90 只)	申请日期(Application date): 2014 年 3 月 1 日
进驻日期(Entering date): 2014 年 8 月 7 日	结束日期(Ending date): 2015 年 12 月 28 日

实验要点，包括实验方法、观测指标、实验结束后处死动物的方法等:

(Outline of experiments, experimental methods, observational index, executing animal method, et. al):

该研究本着实验动物福利和伦理的原则，本实验项目优化设计方案，严格计划动物需要数量，计划需要 90 只 Sprague-Dawley 大鼠。实验方法涉及动物紫外线核黄素交联后的角膜的变化研究。采用氯胺酮和甲苯噻嗪麻醉下行角膜交联术，术后 1、3、7、14、28、42 天分离角膜进行评估。各实验操作均无毒性。实验结束后，麻醉大鼠，使大鼠安乐死，经打包，最后统一焚烧。

In this study, the 90 Sprague-Dawley male rats were used for evaluation of high-intensity corneal collagen cross-linking（CXL） (n=5 each time point). After CXL , the rats were sacrificed by anesthesia with ketamine hydrochloride and xylazine hydrochloride at 1, 3, 7, 14, 28 and 42d. Cornea samples were collected from all rats. After the experiments, all rats were burned.

申请人签名 (Signature of applicant): 朱奕睿 联系电话 (Telephone): 18857743452（653452）

项目负责人签名(Signature of Project director): 陈蔚 联系电话 (Telephone): 13757728118

声明(Statement)：

我将自觉遵守实验动物福利伦理原则，随时接受委员会的监督与检查，如违反规定，自愿接受处罚。

(I will conscientiously abide by the ethical principles of animal welfare, accept the supervision and inspection of the committee at any time, and voluntarily accept the punishment if any infringement.)

项目负责人签名(Signature of Project director)：[illegible]

项目执行人签章(Signature of Project implementation)：[illegible]

审查依据 (Inspection contents)	1. 该项目是否必须用实验动物进行实验，即能否用计算机模拟、细胞培养等非生命方法替代动物或用低等动物替代高等动物进行实验？<br>(Does laboratory animal must be used in the project? Could other methods such as computer simulation, cell cultivation or using the low-grade animal instead of the high-grade animal?)<br><br>2. 表中所填申请人资格和所用动物的品种品系、质量等级、规格是否合适，能否通过改良设计方案或用高质量的动物来减少所用动物的数量？<br>(Are the qualification of applicant，species or strain, grade and specifications of animals suitable? Could the quantity of animals be reduced by improving the study design or using high quality animals?)<br><br>3. 能否通过改进实验方法、调整实验观测指标、改良处死动物的方法，来优化实验方案、善待动物？<br>(Could the study design and animal treatment be refined by ameliorating experimental method, adjusting observational index, executing animal method?)
审查结果 (是否同意申请人的实验方案) (Results of inspection)	实验动物管理和伦理委员会意见 (Attitude of the Animal Management and Ethics Committee)： 同意 (Agree) ☑ 不同意 (Disagree) ☐ 修改后同意（Agree after revised） ☐

伦理委员会主任委员签名(Signature of Ethics Committee Director)：[illegible]

温州医科大学实验动物伦理委员会
温州医科大学实验动物中心
（代章）
(Laboratory Animal Ethics Committee of Wenzhou Medical University & Laboratory Animal Centre of Wenzhou Medical University)
(Stamp)

2015 年 8 月 2 日
